# Nanoarchitectural Control of Nickel-Stabilized Prussian Blue Analogues Improves Hydrogen Peroxide Sensing at Microdisc Electrode Arrays

**DOI:** 10.1021/acsmeasuresciau.6c00131

**Published:** 2026-08-21

**Authors:** Martin L. Zamora, John R. Aggas, Ricardo Matias Trujillo, Mufeeda Mundummal, Khaled Nabil Salama, Anthony Guiseppi-Elie, Rossana E. Madrid

**Affiliations:** † Laboratorio de Medios e Interfases (LAMEIN), Universidad Nacionalde Tucumán Av. Independencia1800, Tucumán, Argentina and Instituto Superior de Investigaciones Biológicas (INSIBIO), CONICET, Chacabuco 461, Tucumán 4000, Argentina; ‡ Center for Bioelectronics, Biosensors and Biochips (C3B), Department of Biomedical Engineering and Department of Electrical and Computer Engineering, 122656 Texas A&M University, College Station, Texas 77843, United States; § Medical Sensing, Kilby Laboratories, Texas Instruments, 13650 N Expy, Dallas, Texas 75246, United States; ∥ Department of Energy and Process Engineering, Norwegian University of Science and Technology, Trondheim NO-7491, Norway; ⊥ Bioengineering Program, Biological, Environmental Sciences and Engineering Division (BESE), King Abdullah University of Science and Technology (KAUST), Thuwal 23955-6900, Kingdom of Saudi Arabia; # Electrical and Computer Engineering Program, Computer, Electrical, and Mathematical Sciences and Engineering Division (CEMSE), King Abdullah University of Science and Technology (KAUST), Thuwal 23955-6900, Kingdom of Saudi Arabia; ∇ Department of Cardiovascular Sciences, Houston Methodist Institute for Academic Medicine and Houston Methodist Research Institute, 6670 Bertner Ave., Houston, Texas 77030, United States; ○ ABTECH Scientific, Inc., Biotechnology Research Park, 800 East Leigh Street, Richmond, Virginia 23219, United States

**Keywords:** hexacyanoferrate, electrodeposition, under-potential deposition, amperometry, biosensors

## Abstract

Hydrogen peroxide (H_2_O_2_) is a central analyte in enzymatic processing, environmental monitoring, and physiological redox signaling, yet its electrochemical quantification remains constrained by limited sensitivity and mediator instability. Here, we demonstrate that purposeful interfacial nanoengineering of a microfabricated microdisc electrode array (MDEA) yields substantial and mechanistically resolvable gains in H_2_O_2_ sensing performance. A Cell-on-a-Chip MDEA (37 Pt microdiscs, 25 μm radius) was modified with nickel-stabilized Prussian blue analogue (FeNiHCF) films deposited using two distinct strategies: (i) potentiostatic, layer-by-layer deposition (FeNiHCF_PS_) and (ii) potentiodynamic codeposition (FeNiHCF_PD_). Although both architectures enhanced charge-transfer kinetics relative to unmodified Pt, their electroanalytical performance diverged markedly. The FeNiHCF_PS_ multilayer conferred 3.6-fold and 2.6-fold increases in H_2_O_2_ sensitivity over the FeNiHCF_PD_ film in the 1–10 μM and 10–100 μM ranges, respectively, and produced up to a 200-fold increase in steady-state current compared with the single-layer architecture. Multiple-scan-rate cyclic voltammetry and electrochemical impedance spectroscopy revealed that these differences arise not from interfacial kinetics alone, but from fundamentally distinct intrafilm charge-transport regimes: FeNiHCF_PS_ films are redox-dense yet transport-limited, whereas FeNiHCF_PD_ films support faster but less extensive charge propagation. When integrated with the radial diffusion advantages of the MDEA geometry, the layered Fe|Ni hexacyanoferrate architecture achieves near-ideal steady-state amperometric behavior at physiologically relevant overpotentials. Characterized using atomic force microscopy (AFM), scanning electron microscopy (SEM), energy-dispersive X-ray spectroscopy (EDX), and X-ray photoelectron spectroscopy (XPS), these findings establish film architecture—not simply composition—as the dominant determinant of mediator performance and provide a generalizable design principle for high-sensitivity, low-overpotential electrochemical peroxide sensing.

## Graphical abstract

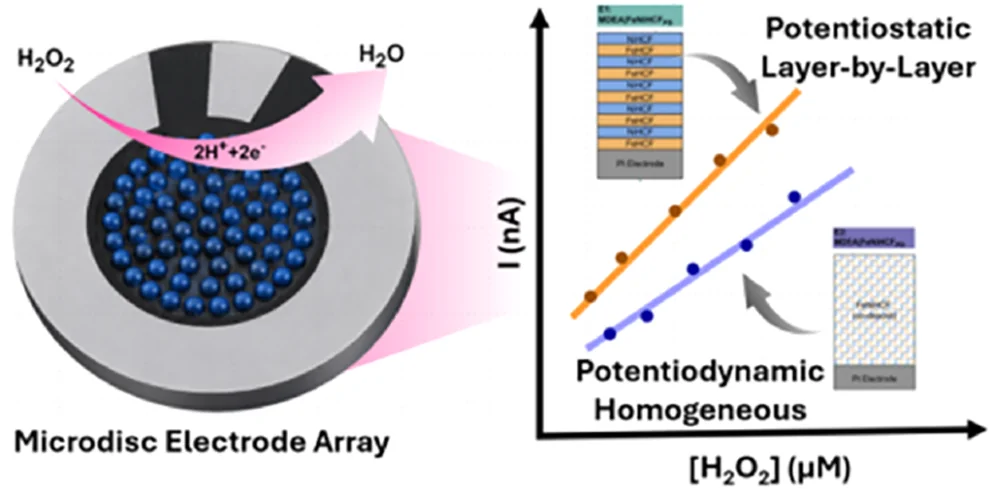

## Introduction

Hydrogen peroxide (H_2_O_2_; 34.01 g/mol) is a small but critically important molecule that functions both as a reactant and as a product in numerous enzymatic and industrial processes. It plays essential roles in food processing, pharmaceutical manufacturing, environmental monitoring, clinical diagnostics, and human physiology. In biological systems, H_2_O_2_ arises as an intermediate during the enzymatic oxidation of organic substrates and is a central member of the reactive oxygen species (ROS) family.^1^ ROS are formed through the incomplete reduction of molecular oxygen and include both radical species—such as superoxide (O_2_^•–^) and hydroxyl radical (^•^OH)—and nonradical species such as H_2_O_2_.^2^ While radical ROS are highly reactive and short-lived, H_2_O_2_ is comparatively stable and membrane-permeable, making it the preferred analyte for assessing oxidative stress and ROS-associated pathologies.^3^

Numerous analytical methods have been developed for H_2_O_2_ quantification,^4^ including chromatography,^5^ titrimetry,^6^ and chemiluminescence.^7^ Although effective, these techniques are often labor-intensive, time-consuming, and require skilled personnel. Electrochemical detection has therefore emerged as a preferred alternative^8^ because of its low instrumentation cost, design flexibility, compatibility with miniaturization, high sensitivity, wide dynamic range, and suitability for point-of-concern monitoring.^9^ Among electrochemical methods for H_2_O_2_ detection, enzymatic biosensors form a distinct subclass in which immobilized enzymes, such as horseradish peroxidase (HRP) and hemoglobin (Hb), facilitate the electrocatalytic reduction of H_2_O_2_. Nevertheless, first-generation (Gen-1) enzyme-based biosensors remain limited by relatively high detection limits and operational constraints. Consequently, significant effort has been directed toward improving sensitivity through advanced electrode architectures and redox mediation strategies characteristic of second-generation (Gen-2) configurations.^10,11^

Prussian blue (PB), a ferric hexacyanoferrate, is among the most widely studied redox mediators for H_2_O_2_ detection. Often referred to as an “artificial peroxidase,” PB catalyzes the reduction of H_2_O_2_ at low overpotentials, similar to horseradish peroxidase (HRP).^12,13^ Its open crystalline lattice selectively accommodates small molecules such as H_2_O_2_, and it can be electrochemically deposited as a dense, redox-active adlayer on electrode surfaces.^14^ However, despite its favorable catalytic properties, PB exhibits limited operational stability. Under cathodic conditions, H_2_O_2_ reduction generates hydroxide ions, creating localized alkaline environments that convert PB to its reduced form, Prussian white, which is partially soluble in aqueous media. This transformation can lead to adlayer dissolution and eventual loss of catalytic function. A recent review, looks critically at how structural, morphological, and defect engineering of Prussian Blue Analogues (PBAs) enhances their performance in electrochemical energy storage and catalytic conversion applications.^15^

In this work, we integrate two complementary strategies to enhance electrochemical detection of H_2_O_2_. First, we employ a microdisc electrode array format that exploits radial diffusion to increase analyte flux and thereby amplify steady-state current. Second, we modify the redox properties of the PB redox layer through nickel incorporation, forming Ni/Fe hexacyanoferrate adlayers designed to improve structural and electrochemical properties. The iron–nickel hexacyanoferrate films were deposited onto the microdisc electrode array using two electrochemical approaches: (i) Potentiostatic layer-by-layer deposition and (ii) potentiodynamic deposition. The atomic composition, morphology, redox, ion transport, and catalytic performance of each configuration were systematically evaluated.

The Electrochemical Cell-on-a-Chip Microdisc Electrode Array has previously been described^16^ as a microlithographically fabricated, dual-analyte electrochemical transducer suitable for amperometric and voltammetric biosensing.^17^ Its fabrication, packaging for potential intramuscular implantation, and electrochemical and impedimetric characterization—with and without hydrogel adlayers—have been reported.^18–20^ The device benefits intrinsically from enhanced sensitivity due to radial diffusion at microdisc electrodes.^21^ However, performance improvements achieved through purposeful nanoengineering of the metal–electrolyte interface have not previously been demonstrated on this platform. Here, nanoengineering refers to the controlled modification of the electrode interface to achieve specific electroanalytical outcomes. The integration of radial diffusion-based microelectrode architecture with stabilized nickel-modified hexacyanoferrate adlayers constitutes the central and original contribution of this study.

## Materials and Methods

### Chemicals and Reagents

The redox mediator layers were prepared from potassium chloride (ACS reagent grade, CAS No.: 7447–40–7; KCl; MW = 74.55 g/mol), nickel­(II) chloride (CAS No.: 7718–54–9; NiCl_2_; MW = 129.60 g/mol), potassium ferricyanide (CAS No.: 13746–66–2; K_3_Fe­(III)­(CN)_6_, MW = 329.24 g/mol), hydrochloric acid (CAS No.: 7647–01–0; HCl; MW = 36.46 g/mol) that were purchased from Sigma-Aldrich Co. (St. Louis, MO, USA) and used as received. Hydrogen peroxide (H_2_O_2_) 30% w/w in water stabilized with dipicolinic acid (5–10 ppm); CAS Number: 7722–84–1; MW = 34.01 g/mol, was purchased from Sigma-Aldrich. This solution is 9.79 M based on a density of 1.11 g/mL at RT. The conductivity of the distilled water used to prepare the solutions was lower than 5 μS/m. Phosphate buffered saline (1× PBS, pH = 7.4) solution (137 mM NaCl, 2.7 mM KCl, 10 mM Na_2_HPO_4_ and 1.8 mM KH_2_PO_4_) was prepared in the standard way. Ferrocene monocarboxylic acid (FcCOOH; CAS# 1271–42–7, MW = 230.05 g/mol), and all other common solvents (dimethylformamide, isopropyl alcohol) were purchased from Sigma-Aldrich Co. (St. Louis, MO, USA) and used as received.

### Microelectrode Array Preparation and Description

The devices used in this work belong to a dual channel microtransducer with double tripolar platinum microelectrodes fabricated on the same device or chip. These are the Electrochemical Cell-on-a-Chip Microdisc Electrode Arrays (ECC MDEA5037-Pt, ABTECH Scientific, Inc., Richmond, VA). Each cell of the device is composed of a Pt working electrode (WE) in the form of a microdisc array of 37 discs, each of 50 μm diameter arranged in a hexagonal close packed configuration with discs that were 100 μm on centers. The cell included a chloridized Ag/AgCl reference electrode (RE), and a Pt counter electrode (CE). The design and fabrication of this electrochemical cell-on-a-chip that employed 10 nm Ti/W adhesion layer and 100 nm of electron beam vapor deposited platinum that was patterned by the lift-off technique and then passivated with 0.5 μm silicon nitride (Si_3_N_4_) that was later fluoro-plasma etched to be windowed to reveal the microdisc array and other electrodes, was fully described elsewhere.^13^ The electrode areas were working microdisc electrode (MDEA = 7.27 × 10^–4^ cm^2^), counter electrode (CEA = 7.27 × 10^–3^ cm^2^) (CE = 100 × WE), and the shared reference electrode (REA = 7.27 × 10^–5^ cm^2^) connected to the five bonding pads. The first MDEA, E1, was potentiostatically modified, yielding FeNiHCF_PS_, hereafter referred to as PS, while the second MDEA, E2, was potentiodynamically modified, yielding FeNiHCF_PD_, hereafter referred to as PD. Each electrode was electrodeposited with its unique form of modified Prussian blue analogue (PBA) using different methods of electrochemical deposition. Figure 1 shows a schematic (1A) and photomicrograph (1B) of the device, an SEM of the various electrodes of the three-electrode electrochemical cell-on-a-chip (1C), details of the dimensions of the microdisc array (1D), the assembly of the device with the portable Pinnacle Dual-potentiostat (1E) and a top down (1F) and side profile (1G) of the radial diffusion at 4 critical times during measurement, derived from COMSOL simulation.

**Figure 1:**
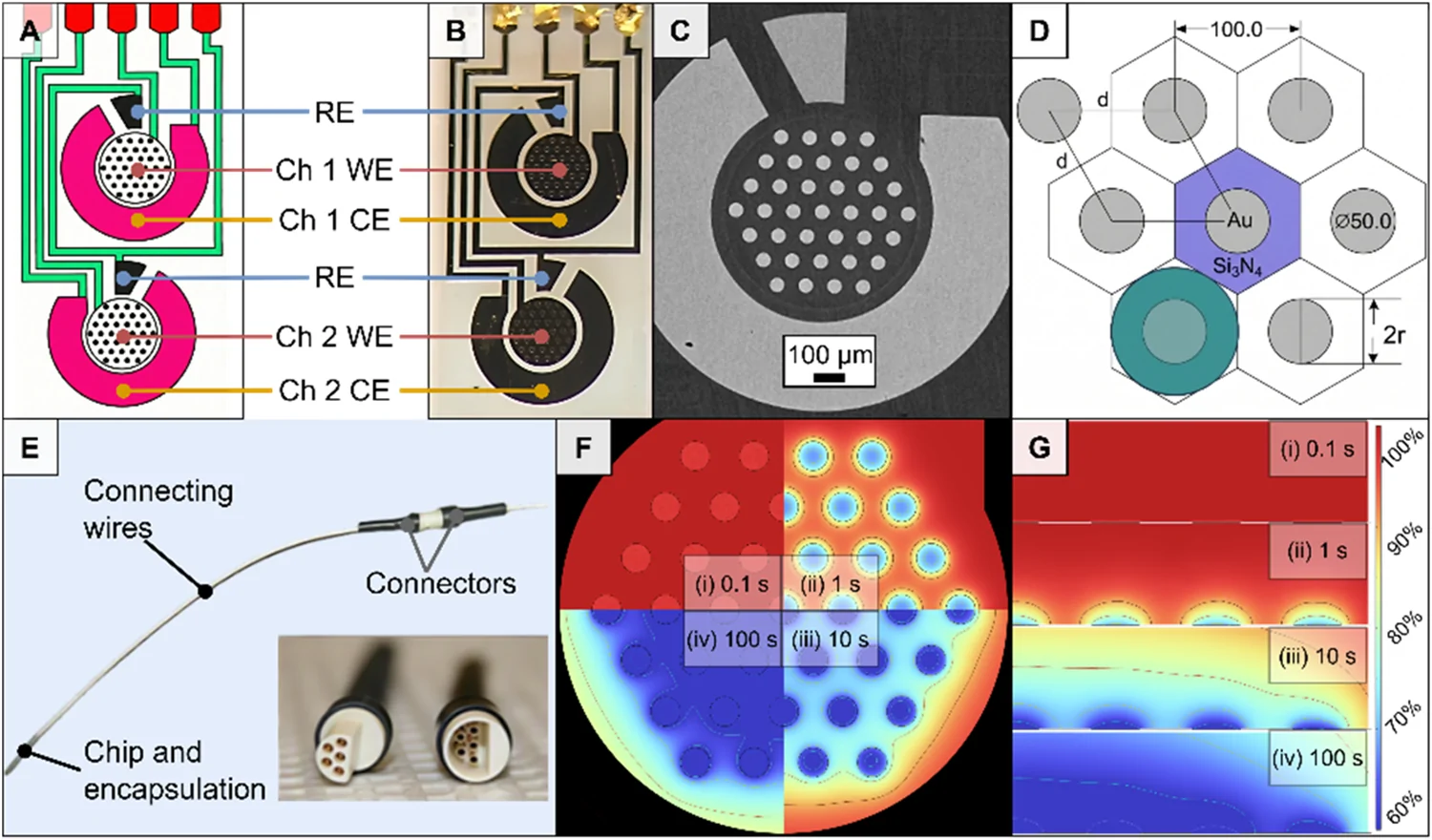
(A) A schematic illustration and (B) labeled photomicrograph of the MDEA 5037-Pt dual responsive transducer. (C) A SEM of a single E-Cell showing the silverized reference electrode. (D) A schematic illustration of the microdisc array. (E) A photograph of the packaged MDEA-5037-Pt chip connected to the Pinnacle 8151 Wireless Dual-potentiostat. An illustration of the three regimes of the temporal development of the diffusion field (F) plan view and (G) cross-sectional view.

### Electrochemical Measurements

Electrochemical measurements were performed both with a dual channel wireless potentiostat, the Pinnacle 8151 Wireless Dual-potentiostat^22,14^ and a Solartron 12508W impedance analyzer system composed of a Solartron 1287 Electrochemical Analyzer and a Solartron 1250 Frequency Response Analyzer, commanded by the software provided by the manufacturer (CorrWare, Scribner Associates Inc.). The individual wells of 96-well plates were used as test containers for sequential electrodeposition and for sequential, quiescent H_2_O_2_ measurements. All the experiments were carried out in phosphate buffer saline solution (PBS), 0.01 M pH 7.2, with KCl (0.1 M) as supporting electrolyte. The cyclic voltammetry was performed between −0.6 and 0.6 V vs reference, for both E1 and E2 devices. For the amperometric measurements, the WEs were polarized at 0.6 V vs reference, for both electrodes (E1 and E2).

#### Potentiostatic Electrodeposition

Electrode E1 was potentiostatically held at −400 mV vs Ag/AgCl and subjected to sequential immersion with controlled timing into four separate aqueous solutions held in the 96-well plate at room temperature:

(A) (5 s) = 50 mM KCl in 10 mM HCl (serving as acidic solvent).
(B) (50 s) = 3.0 mM K_3_Fe­(III)­(CN)_6_ + 3.0 mM FeCl_2_ dissolved in A.
(C) (50 s) = 1.5 mM K_3_Fe­(III)­(CN)_6_ + 3.0 mM NiCl_2_ dissolved in A and finally.
(D) (5 s) = PBS (pH 7.2) (serving as rinse).

The above procedure was repeated five times without interruption, effectively creating a layer-by-layer, multilayered structure of iron (II, III) hexacyanoferrate (II, IIIPB) formed in B followed by a layer of nickel­(II) hexacyanoferrate [Ni­(II)­HCFe] formed in C. The result is E1 = MDEA 5037-Pt|FeNiHCF_PS_ (PS).

#### Potentiodynamic Electrodeposition

The Fe/Ni hexacyanoferrate redox mediator layer was applied to the Pt micro- electrode E2 via 3-electrode potentiodynamic coelectrodeposition using cyclic voltammetry in a freshly prepared deposition solution containing a mixture of 0.5 mM NiCl_2_ and 0.5 mM K_3_Fe­(CN)_6_ in 0.1 M KCl + 0.01 M HCl. A potential sweep between +0.1 and +1.1 V at a scan rate of 100 mV/s for 30 cycles was used, and the modified device rinsed with DI water. The result is E2 = MDEA 5037-Pt|FeNiHCF_PD_ (PD).

### Physical Characterization

Surface morphology and elemental composition of PS and PD films were characterized using scanning electron microscopy (SEM) (Magellan 400 XHR field emission SEM, Thermo Fisher). Elemental composition and spatial distribution of the deposited elements were analyzed by energy-dispersive X-ray (EDX) mapping using a field emission SEM system (Zeiss Merlin FE-SEM, Carl Zeiss). Surface topography was characterized by Atomic Force Microscopy (AFM) using a scanning probe microscopy system (Dimension Icon SPM, Veeco/Bruker). For X-ray Photoelectron Spectroscopy (XPS) (AMICUS, Kratos Analytical, Manchester, UK) measurements a monochromated Al Kα radiation source with a photon energy of 1486.6 eV was employed. Survey spectra were obtained over a binding energy range of 0–1200 eV to confirm the presence of the deposited elements. Subsequently, high-resolution spectra were performed for carbon (C 1s), iron (Fe 2p), and nickel (Ni 2p). Multiple scans were collected and averaged to improve the signal-to-noise ratio. Charge correction was applied using the reference C 1s peak, and background subtraction was performed using a Shirley baseline to obtain a corrected spectrum.

### Transient and Steady-State Amperometric Responses

Two concentration ranges of H_2_O_2_ were evaluated, the lower range from 1 to 10 μM, and the higher range from 10 to 100 μM. Electrodes were stepped from *E* = 0.0|_*t*<0_ to *E* = 0.390|_*t*>0_ V vs Ag/AgCl and the current monitored. The resultant transient and steady state currents were compared against Shoup-Szabo approximations of an ideal MDEA (*N* = 37, *r* = 25 μm, *d*/*r* ≫ 4), a single MDE of equivalent active electrode area (*A*_MDE_ = *A*_MDEA,active_ = 7.27× 10^–4^ cm^2^ ∴*r*_MDE_ = 158 μm), and a single MDE of equivalent total footprint area (*A*_MDE_ = *A*_MDEA,total_ = 2.79× 10^–3^ cm^2^ ∴*r*_MDE_ = 298 μm). The approximations of an MDE, an ideal MDEA (*d*/*r* ≫ 4), and a nonideal MDEA (*d*/*r* ≤ 4) are shown in Table S1.

### Computational Modeling

Computational models of the MDEA 5037 were designed in both 2D and 3D using COMSOL Multiphysics v6.0 run on a PC. 2D and 3D models of the electrochemical cell-on-chip were used to validate electrode geometry and H_2_O_2_ redox performance, optimize sensor methods, and characterize the spatial distribution of electric and chemical transport fields. The geometry of the cell model was prepared according to the schematic shown in Figure 1 and results were normalized by area to allow calculation of kinetic parameters. Computational modeling enabled parametrization to match experimental data, *in silico* optimization of cell design, and prediction of performance characteristics based on design features.

## Results and Discussion

### Electrochemical Characterization of Unmodified Devices

The microfabricated, dual responsive electrochemical-cell-on-a-chip is an electroanalytical device that is based on the microdisc electrode array format. The ECC MDEA 5037-Pt/Au comprises 37 discs each of ϕ = 50 μm diameter and has been previously designed,^23^ microfabricated and packaged,^24^ and characterized^25^ to serve as a biotransducer for the amperometric or voltammetric measurement of analytes such as glucose and lactate that are based on the Generation 1 biosensor configuration.^26,27^ With critical microdisc dimensions of *r* = 25 μm, this device was engineered to work with polymer hydrogel layers that naturally attenuate the diffusivity of small molecules compared to buffer, interstitial fluid or plasma.^28,29^ In this study, the devices are used without hydrogel layers but are otherwise interfacially modified with PB analogues (PBA) and conferred with redox mediation properties.

The two channels of unmodified devices were first characterized by cyclic voltammetry and chronoamperometry in aqueous ferrocene monocarboxylic acid, and the results fitted to simulations reflecting fully developed radial diffusion associated with the microdisc electrode array performance or fully developed semi-infinite linear diffusion associated with planar electrode performance.^30^ The background outlined in the supplemental allows the ratio of the goodness-of-fit to the two extreme models to produce a score reflective of the extent to which the device geometry merited the microarray or planar geometry designation.^12,8^ Figure 2A shows chronoamperograms (potential = 0.0|_*t*<0_, potential = 0.390|_*t*>0_ V vs Ag/AgCl) obtained in 0.125 mM FcCOOH in 1× PBS at RT and the temporal approach to a steady state current under unstirred conditions. Conditions are unstirred to allow the diffusion fields to be developed without forced convection. Both channels of the MDEA 5037 resulted in similar currents measured, which was double the current of an MDE of equivalent active area (MDE_*r*=152 μm_), but less than half of an ideal MDEA. However, the steady state current of both channels aligned with the theoretical current of an MDE of equivalent total footprint area (MDE_*r*=298 μm_), supporting the claim of overlapping diffusion layers at longer times. This can be further understood from a log–log plot of a chronoamperogram in 0.75 mM FcCOOH (Figure 2B). Three separate time regimes can be identified: (i) Purely Cottrellian response (blue), (ii) intermediate diffusion layer overlap (yellow), and (iii) steady state response (green). An accompanying visualization of the diffusion layer at these three separate time regimes illustrate the effect of the growing and eventual overlapping of the diffusion layers on the MDEA (Figure 2C). At the earliest point of the experimental data (*t* = 1 s), the experimental current more closely matches that of the theoretical MDEA_ideal_ (purple line), yet after a short time, the overlap in diffusion layers equates the entire MDEA to that of a single MDE of equivalent total footprint (MDE_*r*=298 μm_, red line). As time goes on and steady state is achieved, the experimental MDEA current continues to follow the theoretical single MDE of equivalent total footprint. Figure 2D shows the experimental calibration plot of *i*_ss_ vs concentration of FcCOOH, again illustrating that the equivalent footprint area MDE approximation is explanatory across the entire measured concentration range.

**Figure 2:**
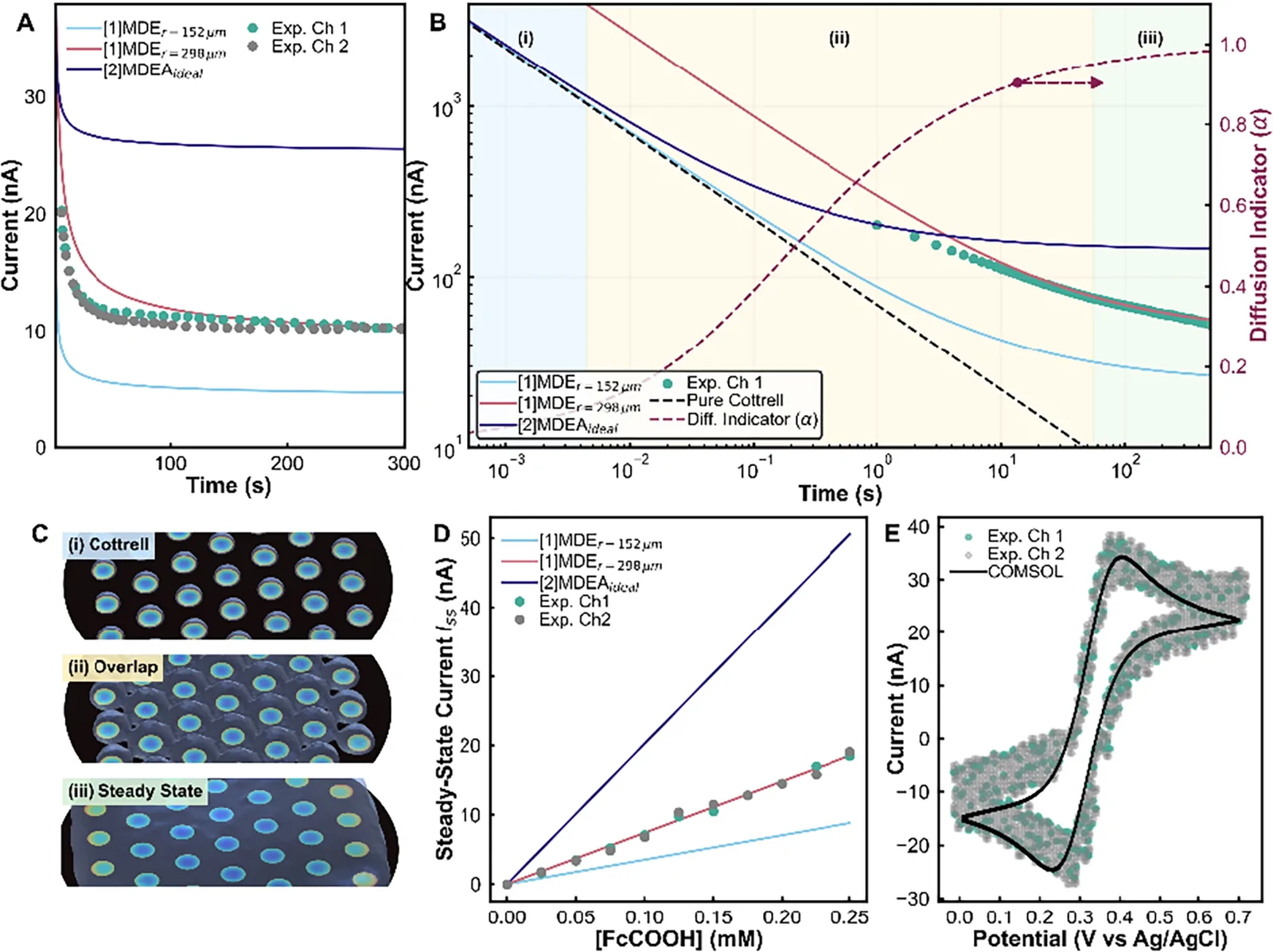
(A) Chronoamperograms (0.0 to 0.390 V vs Ag/AgCl) in 0.125 mM FcCOOH in 1× PBS at RT. (B) Comparison of an experimentally obtained chronoamperograms (green) with those theoretically predicted for an MDE (blue and red), an ideal MDEA (purple), a purely Cottrell response (black dashed) and the diffusion indicator, α, (red dashed). (C) Illustration of the effect of the growing and eventual overlapping of the diffusion layers on the MDEA at the three separate time regimes. (D) Steady state current, *i*_ss_, resulting from chronoamperograms obtained at Ch1 (blue) and Ch2 (red) of the MDEA 5037-Pt as a function of concentration (mM) FcCOOH in 1× PBS at RT and compared to simulations of a single MDE (black) and an ideal MDEA (green) of the same electrode area, and (E) Cyclic voltammograms obtained at 100 mV/s over the range 0–700 mV in 0.125 mM FcCOOH in 1xPBS at RT reveal the peak redox potential be 390 mV.

Lastly, Figure 2E shows the cyclic voltammograms obtained at 100 mV/s over the range 0–700 mV in 0.125 mM FcCOOH in 1× PBS at RT. The redox behavior of FcCOOH was the same at Ch1 and Ch2 of the chip and revealed the peak oxidation potential (*E*_*p*_^*ox*^ = 0.390*V*) vs Ag/AgCl with peak separation, Δ*E*_*p*_ ≈ 110*mV*, being considerably larger than the usual 59 mV associated with ideal behavior. The resultant voltammogram from COMSOL simulation agreed with the experimental data.

### Physical Characterization

#### Structural and Physiochemical Characterization

Scanning electron micrographs (Figure 3A­(i)) show the layer-by-layer PS film to possess a coarse grain structure (Av. = 236 nm) when compared to its coelectrodeposited counterpart, PD film (Av.= 37 nm) (Figure 3B­(i)), with grains that were 6.4 times larger. The root-mean-square surface roughness was calculated to be 18.3 and 16.5 nm, respectively, and was thus of similar roughness, the former being only 1.1 times larger. AFM images and analysis (Figure 3A­(ii), and B­(ii)) confirmed the dramatic difference in the relative coarseness of the grain structures and similar flatness of the two films. EDX elemental survey analyses clearly show the PBA layers were deposited within the discs of the microdiscarray electrodes. Inspection of the EDX distributions of Si (Figure 3A­(iii)), and 3B­(iii), Fe (Figure 3A­(iv) and B­(iv)), Ni (Figure 3A­(v) and 3B­(v)), and EDS layered mapping for C,Fe,Ti,N,Pt,O,Si,Ni (Figure 3A­(vi) and B­(vi)) for the PS layer-by-layer film and the PD clearly show the Si of the silicon nitride passivation layer. When examining PS/PD ratios of Fe_PS/PD_ and Ni_PS/PD_, these were both found to be 1.5, with more Fe and Ni in the PS film than the PD film (Table 1).

**Figure 3:**
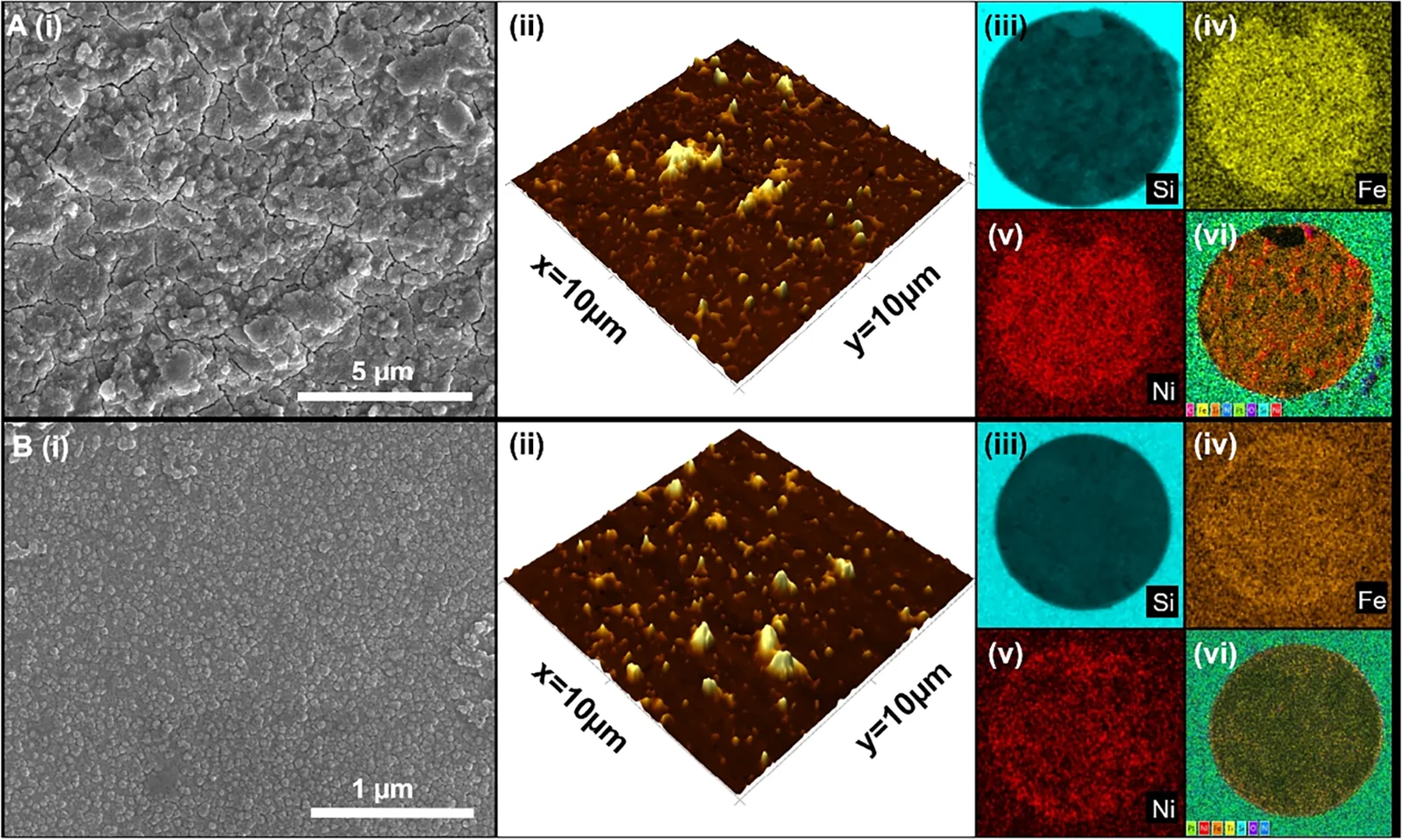
Scanning electron micrographs (i), atomic force micrographs (ii) and EDX distributions of Si (iii), Fe (iv) Ni (v) and EDS layered mapping for C,Fe,Ti,N,Pt,O,Si,Ni (vi) for (A) PS films and (B) PD films.

**Table 1:**
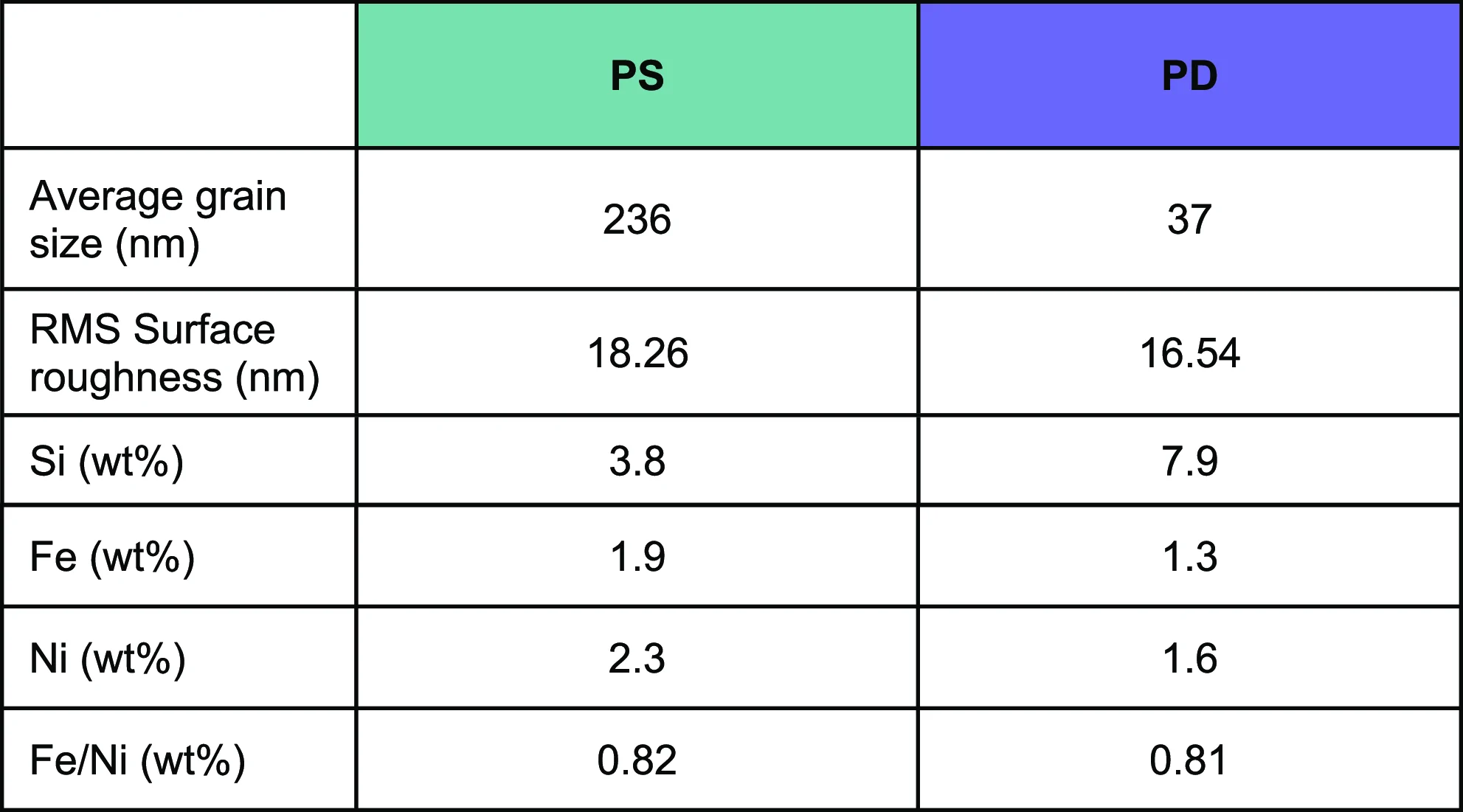
Surface Properties and Elemental Composition Extracted from EDX Analysis of the Electrodeposited PS and PD Films

Figure 4 shows XPS survey-wide spectra (Figure 4A), and high-resolution spectra of Fe 2p (Figure 4B) and Ni 2p (Figure 4C), of the PS and PD films. The survey spectra show the expected elements based on the fabrication processes. Detailed examination of the elemental composition of the PS film using the Fe 2p (10.1 atom %) and Ni 2p (4.1 atom %) peaks, produced an Fe/Ni ratio of 2.5. Likewise, the PD film had Fe 2p (10.0 atom %) and Ni 2p (2.9 atom %) with an Fe/Ni ratio of 3.5. The outer layer of the PS film shows Ni enrichment, consistent with the final layer being formed from 1.5 mM K_3_Fe­(III)­(CN)­6 + 3.0 mM NiCl_2_. When examining PS/PD ratios of Fe_PS/PD_ and Ni_PS/PD_, these were found to be 1.0 and 1.4, respectively, with equal amounts of Fe but Ni enrichment in the PS film compared to the PD-film (Table 2).

**Figure 4:**
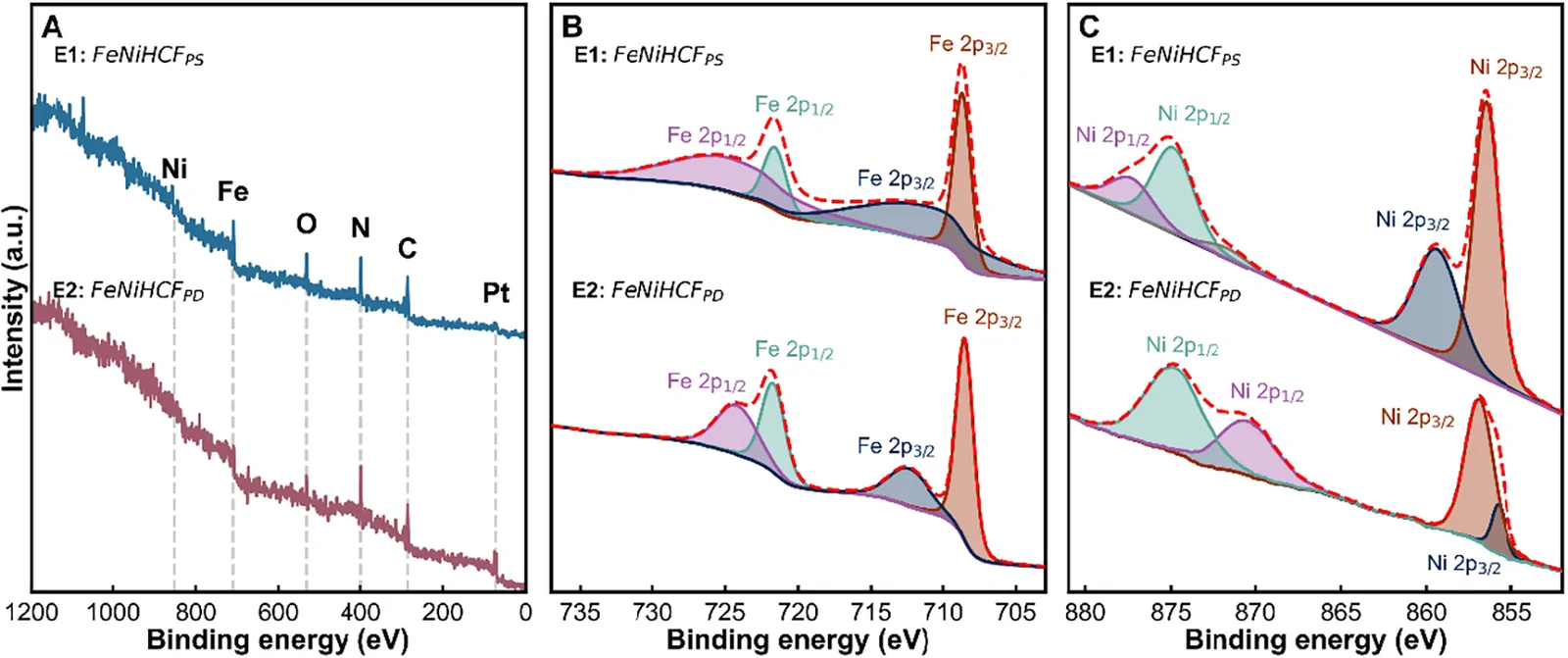
XPS wide spectra (A), high-resolution spectra of Fe 2p (B), and high-resolution spectra of Ni 2p (C) for PS film and PD film.

**Table 2:**
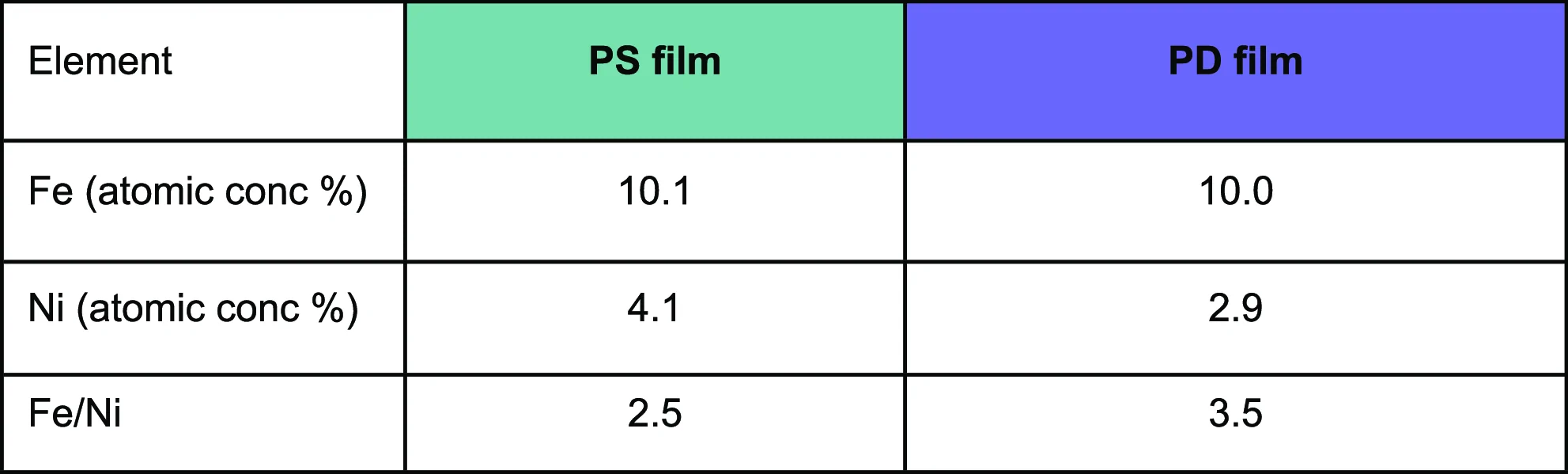
Elemental Quantification Data for Fe and Ni Composition for the Electrodeposited PS and PD Films Using XPS Analysis

### Electrochemical Characterization

A schematic of the possible structure of the PS and PD films is shown in Figure 5A next to an SEM image of an MDEA after electrodeposition. The surface-engineered microdisc array electrodes were evaluated by multiple scan rate cyclic voltammetry (MSRCV) in PBS 7.4 at RT and by AC impedance spectroscopy in PBS 7.4 at RT.

**Figure 5:**
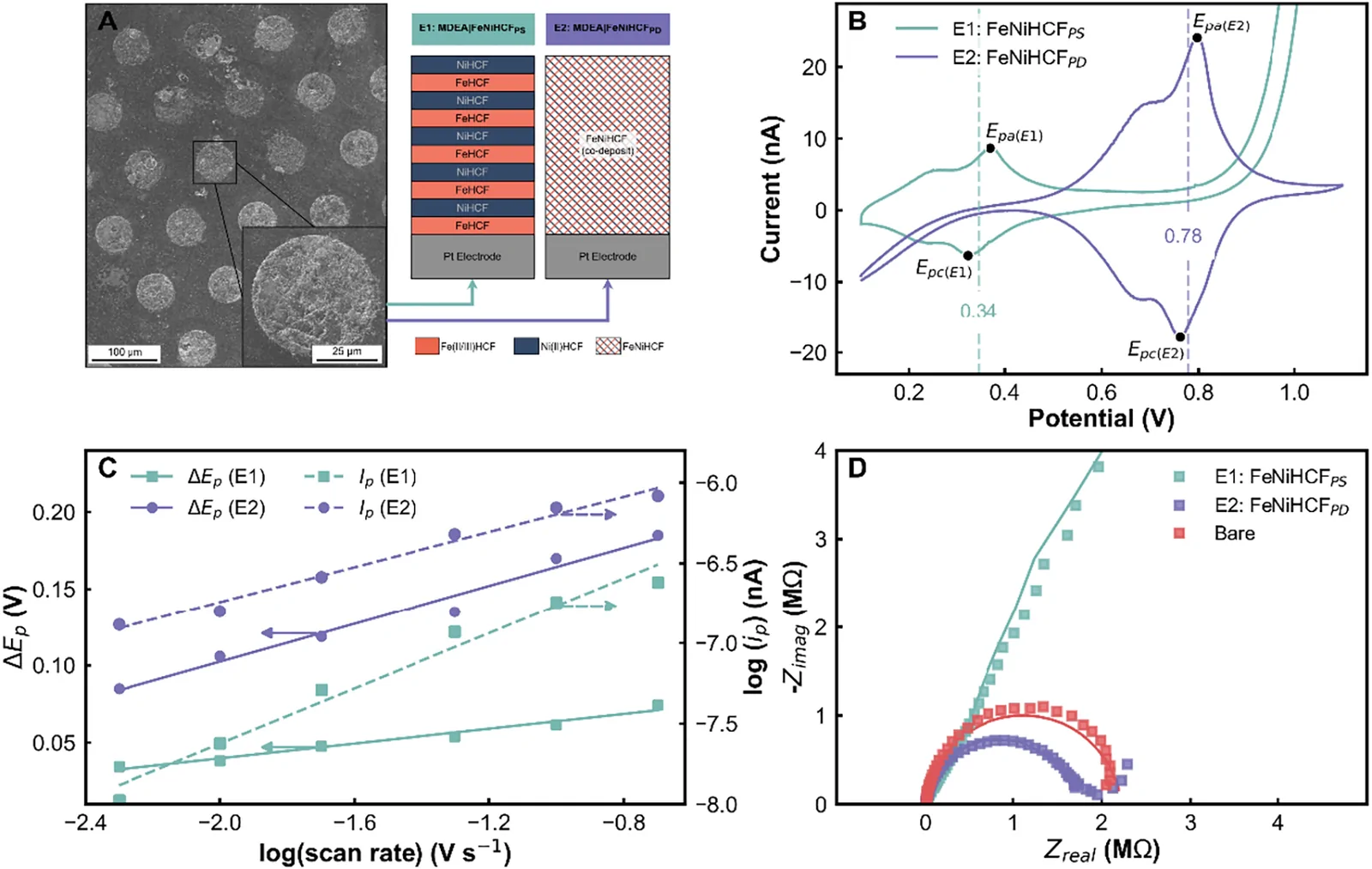
(A) Schematic illustration of the intercalated layers of FeHCF and NiHCF of E1 = MDEA 5037-Pt|FeNiHCF_PS_ and the single layer of mixed FeHCF and NiHCF that comprise the E2 = MDEA 5037-Pt|FeNiHCF_PD_. (B) Cyclic voltammograms of PB modified MDEA 5037-Pt devices in 1× PBS buffer (pH = 7.4) at RT (5 mV/s). (C) Left axis (solid lines): Laviron analysis (ΔEp vs log­(scan rate)). Right axis (dashed lines): peak anodic current vs log­(scan rate) extracted from MSRCV at scan rates of 5, 10, 20, 50, 80, and 100 mV/s. (D) Nyquist impedance spectra of E1, E2, and unmodified MDEA 5037-Pt devices in 1x PBS buffer (pH = 7.4) at RT (measured vs Ag/AgCl at OCP). Points: experimental data, lines: fitted equivalent circuit using R­(QR) for unmodified electrodes and R­(QR)­(QR) for modified electrodes.

#### Cyclic Voltammetry^31^

A representative CV recorded at the slowest scan rate (5 mV/s) is shown in Figure 5B. The two methods of surface engineering revealed dramatically different MSRCV behavior. The PS film produced voltammograms that were *E*_PS_^′^ = 0.34 V vs Ag/AgCl and Δ*E*_*p*_ = 50 mV while the PD film produced voltammograms that were *E*_PD_^′^ = 0.78 V vs Ag/AgCl and Δ*E*_*p*_ = 75 mV. The large difference among the formal potentials has been attributed to the resulting structure pursuant to the mechanism of electrodeposition.^32^ Previous studies have shown that potentiodynamic electrodeposition in electrolytes with potassium cations favors insertion of mobile K^+^ rich (and Nickel poor) layers, which are characterized by more cathodic formal potentials as the ion can stabilize the Fe­(II) form.^33^ Conversely, potentiostatic electrodeposition favors deposition of largely immobile Ni ions in the crystal lattice. The electrodeposition method clearly determines the Prussian blue’s (PB) ability to change to its different analogues. Karyakin^34^ reported that PB exists as a blue inorganic polycrystal. At cathodic potentials, the PB layer will undergo a reduction and discoloration to Prussian white crystals, whereas under more anodic conditions it will change to Prussian green crystals. The voltammograms presented in this work would indicate this behavior.

For an ideal, reversible, surface-confined redox-active film with no accompanying mass transport, the theoretical peak potential separation (Δ*E*_*p*_) is 0 V at slow scan rates. The voltammetric peaks would then be perfectly symmetrical and centered around the formal potential (*E* ^0^′).^35^ Both films show nonzero Δ*V* values even at slow scan rates, suggesting a slow electron transfer rate of a quasi-reversible or irreversible electron-transfer process.^35^ For such processes the rate of heterogeneous electron transfer (*k*_*s*_) is not infinitely fast but finite.^36^ A plot of Δ*E*_*p*_ vs scan rate (log (*v*)) (Laviron method, Figure 5C) shows an increase in the peak separation with an increase of scan rate; with a slower electron transfer rate constant (*k*_*s*_) producing a faster increase of Δ*E*_*p*_ with scan rate.^37,38^ The resultant parameters extracted from the Laviron analysis are shown below in Table 3. These parameters provide insight into how charge may be stored, transported, and structurally accommodated within the two PBA films under physiological electrolyte conditions. The difference in Δ*E*_*p*_ (50 mV vs 75 mV at 100 mV/s) suggests different film thicknesses, structure density, or conductivity, with the larger (75 mV) likely being less conductive, thicker, or a more densely packed layer. The larger *k*_*s*_ indicates stronger electronic coupling between redox centers of the film and the underlying Pt.^36^ Likewise, the smaller apparent surface density of electrochemically addressable redox sites, obtained from low scan rates, suggests lower redox conductivity or higher electron-transfer resistance within that specific film, indicating it is less permeable to ions or has fewer active sites involved in the redox process.

**Table 3:**
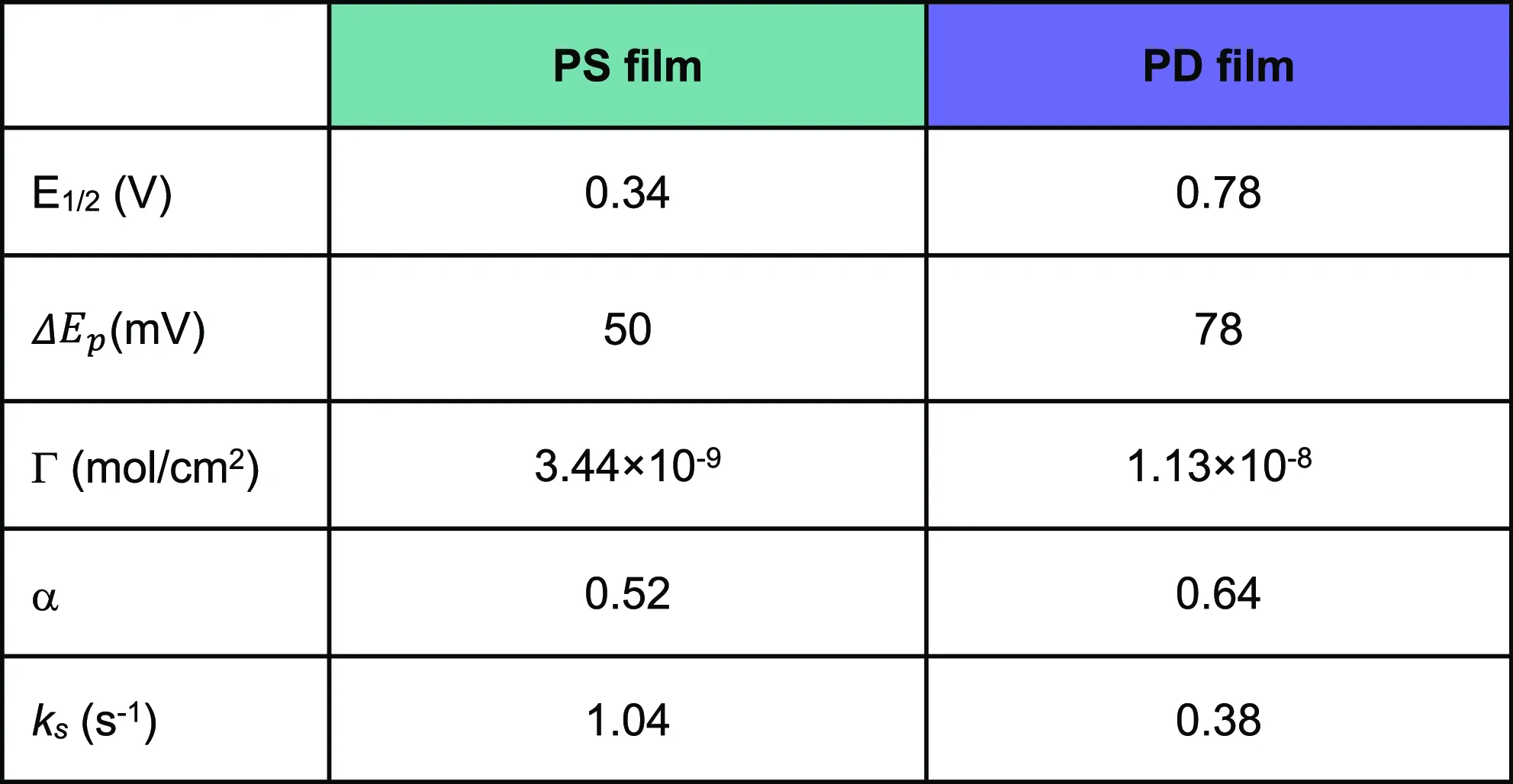
Electrochemical Parameters Extracted from MSCRCV in 1× PBS (pH-7.4) at RT from 0 to 1.1 V vs. Ag/AgCl at 5, 10, 20, 50, 80, and 100 mV/s

The interfacial electron-transfer kinetics that governs electron injection/extraction between Pt ⇄ FeNiHCF redox centers is captured in *k*_*s*_ (s^–1^), which for PS layers is 2.74 times larger than for PD layers. The redistribution of charge, once electrons are injected inside the film, is captured in the calculated effective intrafilm transport parameter, Λ_D_ (cm^2^s^–1^), that informs electron hopping between Fe sites, the density and mobility of compensating ions (Na^+^ /K^+^ /H_3_O^+^), and the structural compliance of the PBA lattice. Λ_S_ was determined by using the Laviron criterion, Λ_D_ = *v*_*c*_(*nF*/*RT*)*L* ^2^, where the critical scan rate, *v*_*c*_, was identified from the onset of scan-rate-dependent peak separation^39^ and the electroactive thickness, *L* = Γ/*C*_redox_, was obtained from the fitted surface coverage, Γ.^40^ This served to establish that Λ_PD_ ≫ Λ_PS_ (Table S2). Accordingly, the PS film has large Γ but small Λ_PS_ which equates to slow charge penetration with only a fraction of surface states used at moderate–to–high scan rates. The PD film has a smaller Γ but larger Λ_PD_ which equates to rapid charge redistribution and near-full utilization of the charge capacity, Γ, over a wider scan-rate window.

Although both films exhibit measurable heterogeneous electron-transfer kinetics at the electrode interface, their dynamic electrochemical behavior is ultimately governed by intrafilm charge propagation. The smaller Λ_PS_ extracted for the PS film indicates transport-limited utilization of redox sites, whereas the larger Λ_PD_ for the PD film enables rapid electron–ion redistribution and sustained reversibility at elevated scan rates. These results decouple interfacial kinetics (*k*_*s*_) from bulk transport (Λ), revealing film architecture as the dominant determinant of dynamic electrochemical performance.

#### AC Impedance Spectroscopy^41^

The resultant Nyquist plots of unmodified MDEA 5037-Pt, MDEA 5037-Pt|PS, and MDEA 5037-Pt|PD in PBS pH 7.4 at *RT* (0 V vs Ag/AgCl) are shown in Figure 5D. The semicircle measured for the unmodified electrode was fit using the simplified Randles equivalent circuit (*R*_S_(*Q*_DL_ *R*_CT_)) to extract the solution resistance (*R*_S_), double layer capacitance (*Q*_DL_), and charge transfer resistance (*R*_CT_) of the Pt|electrolyte interface (Table 4). The high charge transfer resistance (2.13 × 10^6^ Ω) is typical for a measurement where no redox species are involved.^42,43^ Analysis of the electrodeposited films required a more complex equivalent circuit (*R*_S_(*Q*_PBA_ *R*_PBA_)­(*Q*_DL_ *R*_CT_)) to quantify the bulk film properties in addition to electrode/electrolyte properties. The *R*_CT_ of the MDEA 5037-Pt|PS (*R*_CT_ = 2.56 × 10^5^ Ω) showed excellent improvement in charge transfer compared to the bare platinum and roughly 6x lower than its PD counterpart, in rough agreement in the relationship of the *k*_s_ values extracted from the Laviron analysis (*k*_s,PS_ = 1.04 s^–1^, *k*_s,PS_ = 0.38 s^–1^). The layer-by-layer PS film exhibited a highly resistive bulk film resistance (*R*_PBA_ = 2.41 × 10^7^ Ω), 2 orders of magnitude higher than the PD film (*R*_PBA_ = 8.05 × 10^5^ Ω). This high bulk resistance of the PS film is in part a result of the highly coarse and fractured morphology imaged from SEM (Figure 2A­(i)) and the higher average grain size (PS: 452 nm, PD: 39 nm). However, more importantly, bulk resistance is directly associated with the mixed ionic-electronic conductivity of the lattice. As previously discussed, the PS method results in higher immobilized Ni, and less mobile K^+^ in the lattice, while the PD method offers the opposite. The lack of mobile counterions in the PS film, reflected in the 30-fold larger R_PBA_ (Ω), can create a bottleneck in ionic conductivity.

**Table 4:**
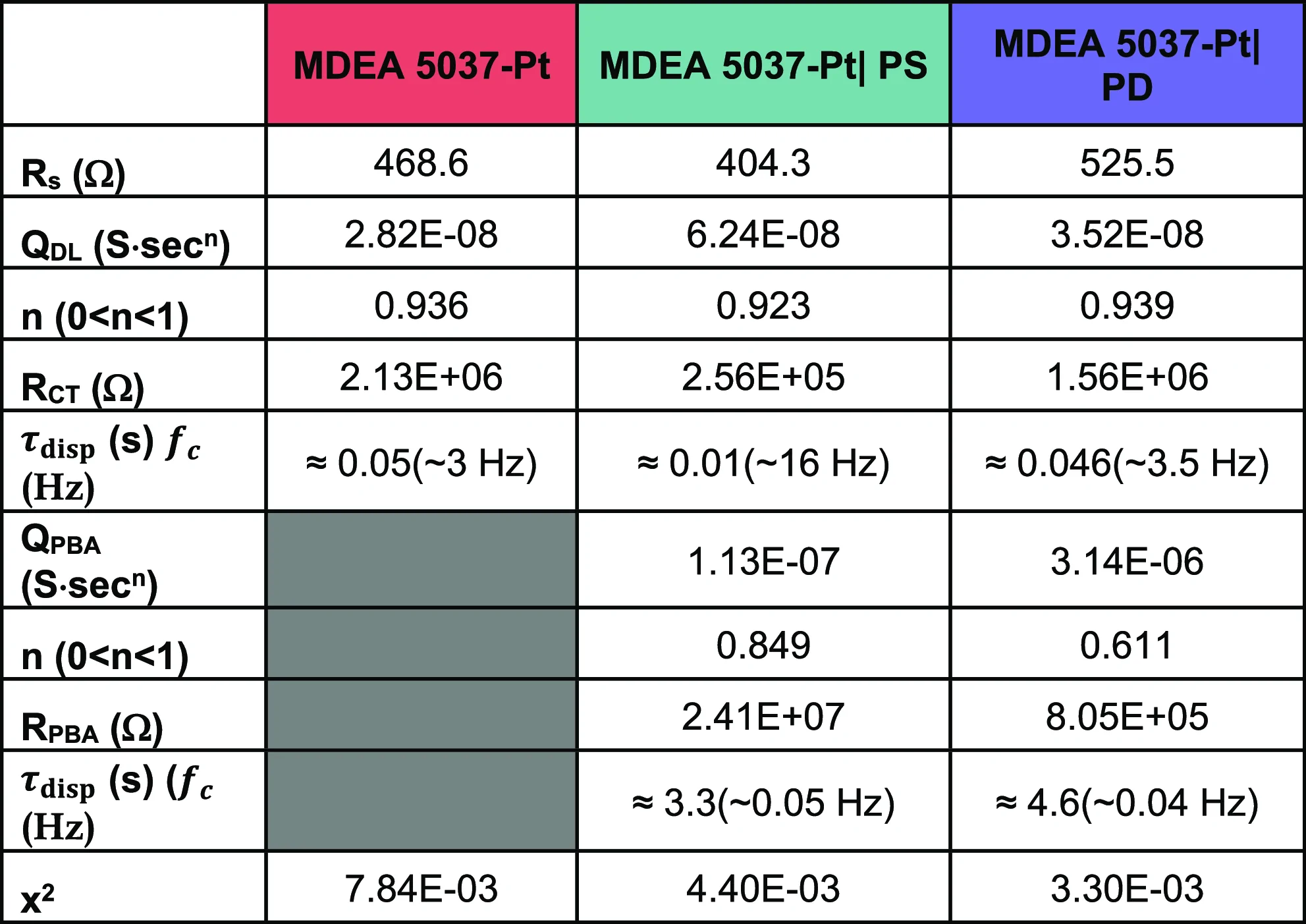
Equivalent Circuit Analysis of Impedance Spectra for the Unmodified MDEA 5037-Pt (*R*_S_(*Q*_CT_ *R*_CT_)) and PBA Modified MDEA 5037-Pt Devices (*R*_S_(*Q*_PBA_ *R*_PBA_)­(*Q*_CT_ *R*_CT_)) in 1× PBS 7.4 Buffer at *RT^a^*

a (*χ* ^2^ = Goodness of Model Fit Parameter).

The five characteristic dispersion times for the three systems, unmodified, PS and PD were estimated using τ_disp_ = (*RQ*)^1/*n*^, while physically meaningful RC time constants could be obtained by converting CPE parameters, *Q*_S_, to effective capacitances, *C*_eff_, using the Brug (interfacial) and Hsu–Mansfeld (PBA) formalisms. The unmodified MDEA exhibits the largest Nyquist semicircle, consistent with a large charge-transfer resistance (*R*_CT_ ≈ 2.1 MΩ) and a near-ideal double-layer CPE (*n* ≈ 0.94), indicative of low electrochemical heterogeneity at the Pt|electrolyte interface. The impedance response is dominated by *a* single interfacial time constant, with no evidence of inductive artifacts. In contrast, PBA-modified electrodes exhibit two distinct time constants, corresponding to the interfacial charge-transfer process and the PBA-controlled transport/redox domain. The PS-modified electrode shows *a* pronounced compression of the charge-transfer semicircle, reflecting *a* substantial reduction in *R*_CT_(∼2.6 × 10^5^Ω). The PBA response is characterized by a moderately nonideal CPE (*n* ≈ 0.85) and an extended low-frequency, diffusion-influenced (Warburg-like) dispersion, consistent with a thick, resistive yet electroactive PBA film that governs the overall impedance at long time scales. The PD-modified electrode displays a strongly nonideal PBA CPE (*n* ≈ 0.61), producing a depressed and broadened low-frequency arc with a substantially lower PBA resistance (*R*_PBA_ ≈ 8.1 × 10^5^ Ω). This behavior is consistent with a highly disordered, diffusive redox network operating under mixed control, with comparable contributions from interfacial charge-transfer kinetics and PBA-mediated transport. Overall, explicit Nyquist decomposition reveals that PS electrodes are predominantly PBA-transport limited, whereas PD electrodes operate under mixed PBA–interfacial control. Importantly, both PBA architectures significantly enhance charge-transfer kinetics relative to the unmodified Pt MDEA.

Laviron’s MSCR-CV analysis probes phenomena of time constant *τ*_*S*_ *≪ t*_CV_ *∼ RT/nFv* while electrochemical impedance spectroscopy and equivalent-circuit analysis probes phenomena of time constant *τ*_S_ *∼ 1/2πf*_*c*_. Taken together, they provide a fully consistent, multiscale picture of charge transfer and transport in the three electrodes. The unmodified MDEA, which exhibits a large Nyquist semicircle with high *R*_CT_ and a near-ideal double-layer CPE, corresponds in the Laviron framework to a system with minimal electroactive coverage and negligible intraelectrode transport effects, such that the response is governed almost entirely by a single interfacial kinetic time constant. Upon incorporation of FeNiHCF films, both synthetic methods independently reveal the emergence of a second, film-controlled process: Laviron analysis captures this through increased apparent redox coverage (Γ) and finite transport limitations, while EIS resolves it as a distinct low-frequency PBA-associated arc. For the PS electrode, the markedly reduced *R*_CT_ but pronounced low-frequency dispersion and moderately nonideal CPE are fully consistent with Laviron’s indication of a redox-rich film with limited internal charge propagation (small Λ), such that intrafilm transport dominates the dynamic response despite efficient electron injection at the interface. In contrast, the PD electrode, which shows a strongly depressed PBA CPE and lower film resistance, aligns with Laviron-derived behavior characteristic of more efficient internal charge redistribution (larger Λ) but lower apparent redox density, yielding mixed control between interfacial kinetics and film transport. Thus, EIS and Laviron analyses converge on the same mechanistic conclusion: PS films are predominantly transport-limited within the PBA phase, whereas PD films achieve faster, more homogeneous charge propagation, even as both architectures substantially enhance charge-transfer kinetics relative to unmodified Pt.

#### Chronoamperometry

Figure 6A shows the calibration plot (0.390 V vs Ag/AgCl) of the unmodified MDEA 5037-Pt, MDEA 5037-Pt|PS and MDEA 5037-Pt|PD at the 2 ranges of H_2_O_2_ tested (0–10 μM and 10–100 μM) in addition to the theoretical ideal MDEA and MDE_r=298 μm_ response. Both the bare and MDEA 5037-Pt|PD illustrated lackluster response to H_2_O_2_, with sensitives lower than that of the equivalent area MDE, being 9.95e-5 and 4.24e-5 μA μM^–1^ over the full con centration range (1–100 μM), respectively. A likely explanation for the diminished response of the MDEA 5037-Pt|PD in H_2_O_2_ is shown in the CV (Figure 5B), where the redox potential of the applied film is clearly higher than that of the potential applied on the electrode, which will result in sluggish behavior. In contrast, the MDEA 5037-Pt|PS resulted in sensitivity (for the high concentration range: 0.144 μAcm^–2^μM^–1^), almost identical to the theoretical MDE response, shown by the parallel slope. The resultant performance parameters of the 3 tested electrodes is shown in the heatmap in Figure 6B, where the performance was compared relative to each metric calculated (green: best performance, red: worst performance). The MDEA 5037-Pt|FeNiHCF_PS_ excelled in most of the individual parameters, except for that of the limit of detection and steady state response time. This is likely due to (1) the obvious background current (*i* = ∼4 nA at 0 μM H_2_O_2_), and (2) a lower charge transfer resistance/lower effective diffusion coefficient of charge within the electrodeposited layer.

**Figure 6:**
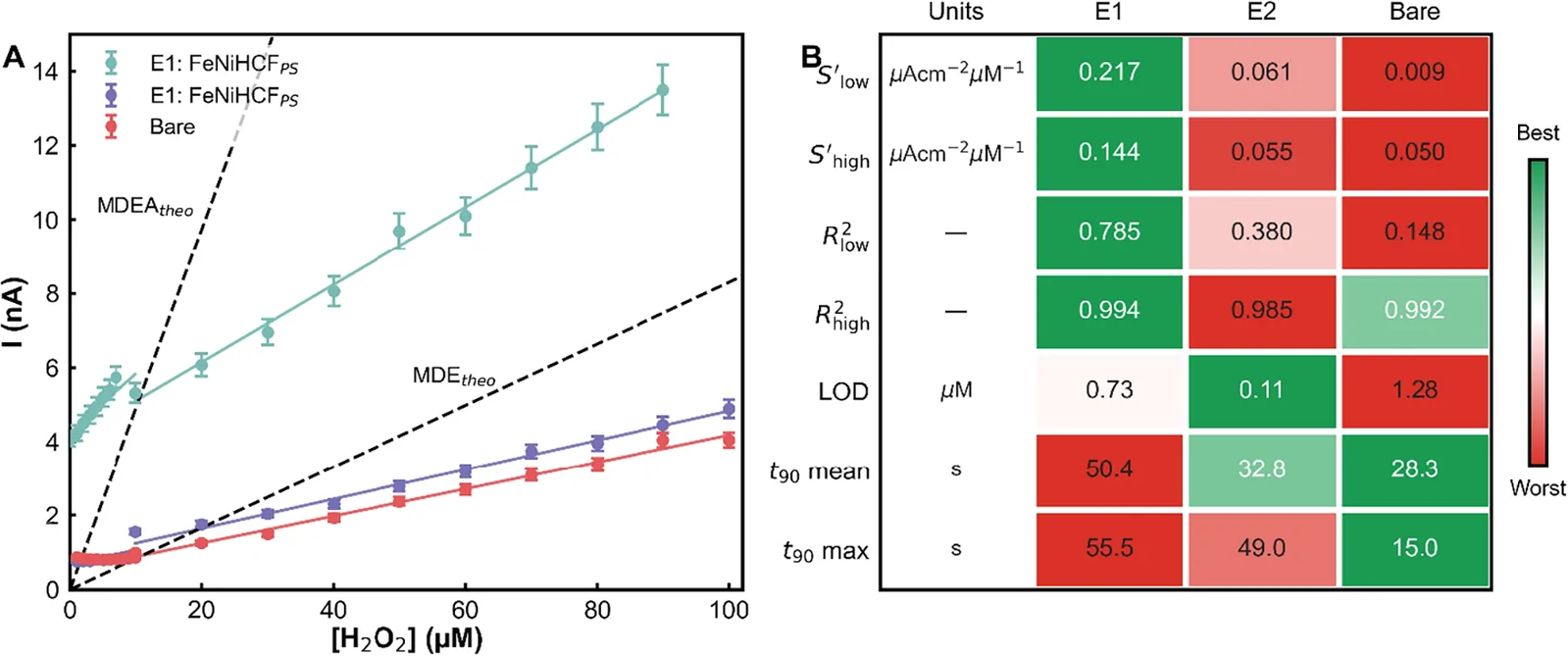
Plots of the steady state current at the unmodified, potentiostatically modified device E1 = MDEA 5037-Pt|PS device, and of the potentiodynamically modified device E2 = MDEA 5037-Pt|PD in varying concentrations of H_2_O_2_ in 1x PBS at RT, showing: (A) Over the low (0 −10 μM H_2_O_2_) and high (10 – 100 μM H_2_O_2_) concentration ranges, and (B) the resulting heatmap of sensitivities (green: best, red: worst).

To further provide context for this work we present a comparison of the performance of the presently prepared PS and PD films on the MDEA-Pt electrodes with performance of recently published Prussian blue analogues using various electrode configurations. Table 5 compares the sensitivity (*S* = d*R*/d*C*) (which may be normalized to per unit active area (*S*_*A*_) to enable fair comparison across sensor geometries), limit of detection (LoD = 3σ/*S*), and dynamic range (Log_10_(*C*_max_ /*C*_min_)) of several, select, recently published PBAs. Moreover, we include the electrode material and electrode design.

**Table 5:** A Comparison of the Performance of the Presently Prepared PS and PD Films on the MDEA-Pt Electrodes with Performance of Select, Recently Published Prussian Blue Analogues on Various Electrode Configurations^a^

refs	PBA	electrode/material	electroactive area (cm^2^)	raw sensitivity (μA μM^–1^)	area-normalized sensitivity *S*_*A*_ (μA μM^–1^cm^–2^)	LoD (μM)	dynamic range	NIR_*A*_
Chen, 2024 ^44^	WS_2_–PBA	Carbon/SPE	0.070	0.045	0.640	0.17	3.38	12.8
Promsuwan, 2025 ^45^	PEDOT–PB	MDE/GC	0.031	0.037	1.190	0.06	3.60	71.4
Fernández, 2022 ^46^	PB-fCNT/TiO_2_.ZrO_2_ (36)500 °C.20/GC	MDE/GC	0.196	0.018	0.092	1.8	3.00	0.15
Gayda, 2021 ^47^	gCuHCF	MDE/Gr	0.126	0.045	0.360	0.30	3.00	3.6
Barber, 2023 ^48^	PB-CS	IDE/LIG	0.283	0.061	0.220	6.31	2.00	0.07
Tehrani, 2021 ^49^	PB/3D-PC	MDEA/PC	0.050	0.024	0.480	0.22	3.27	7.1
This work	FeNiHCF_Ps_	MDEA/Pt	0.000727	9.95 × 10^–5^	0.137	0.73	1.00	0.19
This work	FeNiHCF_PD_	MDEA/Pt	0.000727	4.24 × 10^–5^	0.058	0.11	1.00	0.53

a WS_2_ = Tungsten Disulfide; PEDOT = Poly­(3,4-ethylenedioxythiophene); fCNT = Functionalized Carbon Nanotube, gCuHCF = Green-synthesized Copper Hexacyanoferrate; PB-CS= Prussian Blue Chitosan Composite; SPE = Screen-Printed Electrode; MDE = Microdisc Electrode; IDE = Interdigitated Electrode; LIG = Laser-Induced Graphene (Graphitic Carbon); MDEA = Microdisc Electrode Array; GC = Glassy Carbon; Gr = Graphite; Pt = Platinum; PC = Pyrolytyc carbon.

While highly competitive, but not outstanding in any given metric, deeper analysis of our sensors’ performance may be gleaned from the unified figure of metric (FOM), the Normalized Information Responsivity (NIR). NIR is based on Fisher information theory^50,51^ and the Cramér–Rao Lower Bound (CRLB)^52^ and seeks to quantify how much statistically resolvable information a sensor can extract about analyte concentration across its operational range. The NIR value shifts the focus from purely electrical or physical metrics to the amount of extractable information a sensor can produce.^53,54^

1 $${\mathrm{NIR}}_{A}=\frac{S_{A}}{\mathrm{LoD}}{\mathrm{Log}}_{10}\left(\frac{C_{\max}}{C_{\min}}\right)$$

Higher NIR values reward sensors that combine high gain, low detection thresholds, and broad operational spans. Accordingly, a high NIR corresponds to strong noise-normalized sensitivity maintained across a wide concentration range. NIR therefore provides a unified framework for benchmarking sensor performance, enabling cross-platform comparison and informed evaluation of design trade-offs in sensor development. Our PS and PD sensors sit in the middle of the range of recently studied PBA analogues with PD being superior to PS despite its lower sensitivity.

## Conclusions

The microlithographically fabricated microdisc electrode array format of ϕ=50 μm with 100 μm on centers was investigated for enhanced redox response arising from radial diffusion. The MDEA 5037-Pt device design was found to be ∼ 50% efficient compared to an ideal MDEA but 370% more efficient than a planar electrode at mid time regimes. Microdiscs were surface modified with two types of enhanced PB analogue nano films, each by a unique electrodeposition method, the layer-by-layer PS film and the homogeneously mixed PD film.

PS films were richer in both Fe and Ni, with the Fe/Ni ratio being lower in PS (2.5) than in the PD film (3.5), confirming more Ni in the PS films. Both films were of similar RMS surface roughness, but showed remarkably different scan-rate voltammetric behavior, with PS films being quasi-reversible. The method of Laviron revealed the PS film to be more electronically coherent, chemically uniform, and lattice-ordered; while the PD film was electronically resistive, more heterogeneous and/or defect-mediated. Although the PS film exhibited substantial electroactive coverage, its smaller Γ value reveals a kinetically constrained internal transport network. In contrast, the PD film supported more rapid electron–ion propagation, consistent with a more open and dynamically accessible framework. Although both films exhibit measurable heterogeneous electron-transfer kinetics at the electrode–film interface, their dynamic electrochemical behavior is ultimately governed by intrafilm charge propagation. The smaller Γ extracted for the PS film indicates transport-limited utilization of redox sites, whereas the larger Γ for the PD film enables rapid electron–ion redistribution and sustained reversibility at elevated scan rates. These results decouple interfacial kinetics (*k*_*s*_) from bulk transport (Γ), revealing film architecture to be the dominant determinant of dynamic electrochemical performance.

Potentiostatically electrodeposited layer-by-layer of alternative iron hexacyanoferrate (FeHCFe) and nickel hexacyanoferrate (NiHCFe) produced films (PS) that conferred 3.6 and 2.6 times more sensitivity to H_2_O_2_ in the 0–10 μM and the 10–100 μM range, respectively, than the mixed nickel/iron hexacyanoferrate (PD) prepared by potentiodynamic electrodeposition. Moreover, the PS film was able to follow the i_*ss*_ model at high H_2_O_2_ concentration. It can be inferred from these results that the alternating Fe|Ni layered structure likely established domains that favored improved electron transfer in response to H_2_O_2_. Viewed through the lens of the NIR FoM the PD films produced more information rich hydrogen peroxide sensors despite its lower sensitivity.

## Supplementary Material



## Data Availability

The data of this work are available upon reasonable request to the corresponding authors.

## Notes

Details for the transient and steady-state responses and the relevant equations, diffusion field, and effective behavior for MDE, an ideal MDEA (*d*/*r* ≫ 4), and a nonideal MDEA (*d*/*r* ≤ 4), (ii) the approach to calculate the surface diffusion coefficient for the two Prussian blue analogue (PBA) films, and (iii) the approach to calculate the Normalized Information Responsivity (NIR) (PDF)
