# Supplementary material for: Nanoarchitectural Control of Nickel-Stabilized Prussian Blue Analogues Improves Hydrogen Peroxide Sensing at Microdisc Electrode Arrays
Source: ACS Meas Sci Au. 2026 Aug 21;6(5):1624–35. doi: 10.1021/acsmeasuresciau.6c00131 (PMC13693283; doi:10.1021/acsmeasuresciau.6c00131)
Supplement: Supplementary file 1 [file tg6c00131_si_001.pdf]

Nanoarchitectural Control of Nickel-Stabilized Prussian Blue Analogues Improves Hydrogen Peroxide Sensing at Microdisc Electrode Arrays (Supplemental)

Martin L. Zamora[1], John R. Aggas[2,3], Ricardo Matias Trujillo[1,4], Mufeeda Mundummal[5], Khaled Nabil Salama[5,6], Anthony Guiseppi-Elie[2,7,8,*], Rossana E. Madrid[1,2,*]

[1]Laboratorio de Medios e Interfases (LAMEIN), DBI, FACET, Universidad Nacional de Tucumán Av. Independencia 1800, Tucumán, Argentina and Instituto Superior de Investigaciones Biológicas (INSIBIO), CONICET, Chacabuco 461 Tucumán, Argentina.

[2]Center for Bioelectronics, Biosensors and Biochips (C3B®), Department of Biomedical Engineering and Department of Electrical and Computer Engineering, Texas A&M University, College Station, TX 77843, USA

[3]Medical Sensing, Kilby Labs, Texas Instruments, 13650 N Expy, Dallas, TX 75246, USA

[4]Department of Energy and Process Engineering, Norwegian University of Science and Technology, Trondheim, NO-7491, Norway

[5]Bioengineering Program, Biological, Environmental Sciences and Engineering Division (BESE), King Abdullah University of Science and Technology (KAUST), Thuwal 23955-6900, Kingdom of Saudi Arabia

[6]Electrical and Computer Engineering Program, Computer, Electrical, and Mathematical Sciences and Engineering Division (CEMSE), King Abdullah University of Science and Technology (KAUST), Thuwal, 23955-6900 Kingdom of Saudi Arabia

[7]Department of Cardiovascular Sciences, Houston Methodist Institute for Academic Medicine and Houston Methodist Research Institute, 6670 Bertner Ave., Houston, TX 77030, USA.

[8]ABTECH Scientific, Inc., Biotechnology Research Park, 800 East Leigh Street, Richmond, VA 23219, USA

*Corresponding authors

guiseppi@aiimsei.org (A. Guiseppi-Elie, Tel.: +1(804)347.9363)

rmadrid@herrera.unt.edu.ar (R. E. Madrid, Tel.: +54 9 381 415-2130)

Transient and steady-state responses

For a single microdisc electrode (MDE) of radius r, the Shoup-Szabo approximation has been successfully used to model the transient response, expressed as *i(t)* as shown in **Table S1**, where *n* is the number of electrons transferred, *F* is the Faraday constant, *D* is the diffusion coefficient, *C* is the bulk concentration of electroactive species, and τ and $f(\tau)$ are defined in the last row of the table. [1-3] The $f(\tau)$ captures the transition from short-time planar diffusion to long time hemispherical diffusion, ultimately leading to a steady state current (i_{ss}). [4, 5]

A key aspect of microdisc electrode arrays (MDEAs) is the center-to-center spacing (d) of the individual microdiscs and their relationship to the radius (r) of each microdisc. [6] In order to achieve true, unadulterated radial diffusion at all reasonable time scales during measurement (resulting in the highest possible signal gain per active area), the relationship between the center-to-center distance (d) of each disc and the disc radius (r) should follow. [3, 7]

$$\frac{d}{r} \gg 4 \qquad \textit{Eq. S1}$$

This rule ensures neighboring diffusion layers remain independent, and the resultant temporal and steady state current measured at an MDEA would be a current scaled by the number of microdiscs (N), shown in the 2nd column of **Table 1**. In cases where the d/r ratio does not satisfy this rule, the Shoup-Szabo approximation only applies for times in which the diffusion layers of the individual microdiscs have not yet overlapped:

$$t \ll \frac{4r^2}{\pi D} \qquad \textit{Eq. S2}$$

At longer times, the Shoup-Szabo approximation increasingly overestimates the current (points to graph B, ideal MDEA), as the diffusion layers have overlapped and the effective gain of N microdiscs operating under radial diffusion has diminished (**Eq. S3**). As such, the resultant i_{ss} resembles that of a single MDE (**Eq. S4**), but with a scaling of the square root of the number of discs:

$$i_{ss,MDEA} = 4\sqrt{N}nFDCr \qquad \textit{Eq. S3}$$

$$i_{ss,MDE} = 4nFDCr \qquad \textit{Eq. S4.}$$

The shift in current response from short times (Cottrell-like) where each microdisc has an independent diffusion zone to longer times where diffusion zones overlap has been characterized by, using a diffusion indicator, α (**Eq. S5**): [7]

$$\alpha = 2\,\frac{d(log(J))}{d(log(\tau))} + 1 \qquad \textit{Eq. S5}$$

where J is the dimensionless current (*J=I/(FrDc)*). This behavior is illustrated in **Fig. S1**, which plots the resultant COMSOL simulations of current of an idealized N microdisc electrode array, where d/r>>4 (blue), a single microdisc electrode of equivalent geometric area of the sum of the N microdiscs (green), a single microdisc electrode of equivalent total footprint of the array lattice of the MDEA (red), and the MDEA 5037 (orange).

Distinct regimes are identified, showing that at extremely short time frames (typically < 0.001 s) the MDEA 5037 (d/r=4) operates as N individual microdiscs with Cottrell-like planar diffusion behavior (regime I). As time progresses, the diffusion layer thickness becomes comparable to the radius of each microdisc, resulting in independent radial diffusion at each microdisc. During these short time frames, the Shoup-Szabo approximation (**Eq. of Table 1**) applies. However, beyond this point in time, as the diffusion layer continues to grow, the current response will deviate from the Shoup-Szabo approximation (regime III). In this time period, there is a transition from the MDEA acting as N individual microdiscs, to one microdisc of equivalent footprint, where the steady state current (regime IV), will still be higher than that of a single microdisc of equivalent area (green), but lower than that of the MDEA where d/r>>4 (blue). Thus, from the theoretical current vs. [H_2O_2] dashed lines shown in **Fig. 4B**, the resultant current of the potentiostatically deposited film lies between these two theoretical limits, consistent with the computational model which predicts a steady state current for MDEA-5037 between the equivalent area microdisc (green) and the idealized independent disc MDEA (blue). The approximations of an MDE, an ideal MDEA (d/r >> 4), and a non-ideal MDEA (d/r <= 4) are shown in **Table S1**.

Table S1. Relevant Equations, Diffusion Field, and Effective Behavior for MDE, an Ideal MDEA (d/r>>4), and a Non-ideal MDEA (d/r<=4)

	**MDE**	**MDEA**	**MDEA**
**Electrode geometry**	r	$\frac{d}{r} \gg 4$	$\frac{d}{r} \leq 4$
$\mathbf{i(t)}$	$i(t) = 4nFDCrf(\tau)$	$i(t) = 4NnFDCrf(\tau)$	$i(t) = 4\sqrt{N}nFDCrf(\tau)$
$\mathbf{i_{ss}}$	$i_{ss} = 4nFDCr$	$i_{ss} = 4NnFDCr$	$i_{ss} = 4\sqrt{N}nFDCr$
**Diffusion**	Hemispherical	Independent radial diffusion zones	Overlapping diffusion zones
**Effective Behavior**	Single microdisc	N independent microdiscs	Equivalent macrodisc $r_{eff} = \sqrt{N}r$
$f(\tau) = 0.7854 + \frac{0.8862}{\sqrt{\tau}} + 0.2146\exp\left(-\frac{0.7823}{\sqrt{\tau}}\right)$			$\tau = \frac{Dt}{r^2}$

To calculate the surface diffusion coefficient, Λ_D, for the two Prussian blue analogue (PBA) films:

*Calculate Electroactive Film Thickness (**L**)*

Define L

$$L = \Gamma / C_{redox} \qquad \text{Eq. S6}$$

We first estimate the redox site concentration for a typical FeNiHCH film using the lattice parameter of 10.2 Å and density of 1.8 g/cm^3 which yields the molar volume of 185 cm^3/mol [8] and correspondingly a C_{redox} of 5.4 x10-3 mol/cm^3.

For FeNiHCF$_{PS}$ $L = \Gamma/C_{redox} = (3.44\,\text{x}10^{-9})(5.4\,\text{x}10^{-3}) = 6.4\,nm$

For FeNiHCF$_{PD}$ $L = \Gamma/C_{redox} = (1.13\,\text{x}10^{-8})(5.4\,\text{x}10^{-3}) = 20.9\,nm$

Although PD films are 3.3 times thicker than PS films, they are both sufficiently thin to justify the Laviron thin film analysis.

*Identify the Critical Scan Rate (**ν_c**)*

The critical scan rate $\nu_c$denotes that scan rate where the peak separation ΔE_pbegins and is denoted by a match of the experimental time scale with the kinetic timescale

$(nF\nu/RTk_s) \approx 1 \; or \; (\nu_c = k_s \, RT/nF)$ *Eq. S7*

For $FeNiHCF_{PS}$ $\nu_c = (1.04 \text{ s}^{-1})(0.0257 \text{ V}) = 0.0267 \text{ Vs}^{-1} = 26.7 \, mVs^{-1}$

For $FeNiHCF_{PD}$ $\nu_c = (0.38 \text{ s}^{-1})(0.0257 \text{ V}) = 0.0098 \text{ Vs}^{-1} = 9.8 \, mVs^{-1}$

Calculate $(\boldsymbol{\Lambda_D})$

Using the formula

$\Lambda_D = \nu_c \, (nF/RT) \, L^2$ *Eq. S8*

For $FeNiHCF_{PS}$ $\Lambda_D = \left(0.0267 \text{ Vs}^{-1}\right)(38.92)(6.36 \text{ x}10^{-7})^2 = 4.21 \text{ x}10^{-13} cm^2 s^{-1}$

For $FeNiHCF_{PD}$ $\nu_c = \left(0.0098 \text{ Vs}^{-1}\right)(38.92)(2.09 \text{ x}10^{-6})^2 = 1.66 \text{ x}10^{-12} cm^2 s^{-1}$

These calculated diffusion coefficients fall within the expected range ($1 \text{ x}10^{-10} - 1 \text{ x}10^{-14} cm^2 s^{-1}$) for Prussian blue analogue films. [9]

Table S2. Calculated Parameters Based on MSCRCV Data in 1xPBS (pH-7.4) at RT.

	MDEA 5037-Pt| $FeNiHCF_{PS}$	MDEA 5037-Pt| $FeNiHCF_{PD}$
L(nm)	6.4	20.9
ν_c(mV s^{-1})	26.7	9.8
Λ_D(cm^2 s^{-1})	4.21×10^{-13}	1.66×10^{-12}

Introducing the Normalized Information Responsivity (NIR)

The Normalized Information Responsivity (NIR) provides a quantitatively meaningful, information-centric metric for comparing sensor performance. Rather than emphasizing any single attribute, NIR reflects a sensor's capacity to produce resolvable and reproducible signal changes across its usable operating range. As such, it is particularly well suited for benchmarking competitive sensors in analytical electrochemistry and biosensing.

NIR is formally traceable to Fisher information theory and the Cramér–Rao Lower Bound

(CRLB). [10-12] Under the CRLB, the minimum achievable variance in concentration estimation scales inversely with S^2/σ^2, directly linking noise-normalized sensitivity to estimation precision. Although Fisher information is formally defined locally in parameter space, NIR aggregates this behavior over the sensor's calibrated concentration range to yield a practical, system-level figure of merit. In this sense, NIR provides a finite-domain approximation to the Fisher information accumulated across the sensor's effective operating range.

By explicitly normalizing sensitivity to baseline noise and scaling this quantity by the usable concentration span, NIR approximates the total statistically resolvable information available for analyte concentration estimation. Unlike isolated metrics such as sensitivity or limit of detection alone, NIR captures the cumulative analytical information a sensor can extract across its operational domain. The resulting scalar is dimensionless, enabling direct comparison across sensor modalities and transduction mechanisms.

NIR quantifies the amount of statistically resolvable information a sensor can extract about analyte concentration over its operational range and is calculated from the sensitivity $S = dR/dC$(which may be expressed per unit active area to enable fair comparison across sensor geometries), the dynamic range expressed in decades as $\log_{10}(C_{\max}/C_{\min})$, and the limit of detection defined as $\mathrm{LoD} = 3\sigma/S$:

$$NIR = \frac{S}{LoD} log_{10}\left(\frac{C_{max}}{C_{min}}\right) \qquad \textit{Eq. S9}$$

Higher NIR values reward sensors that combine high gain, low detection thresholds, and broad operational spans. Accordingly, a high NIR corresponds to strong noise-normalized sensitivity maintained across a wide concentration range. NIR therefore provides a unified framework for benchmarking sensor performance, enabling cross-platform comparison and informed evaluation of design trade-offs in sensor development.

References

(1) Lee, H. J.; Beriet, C.; Ferrigno, R.; Girault, H. H. Cyclic voltammetry at a regular microdisc electrode array. *Journal of Electroanalytical Chemistry* **2001**, *502* (1), 138–145. DOI: https://doi.org/10.1016/S0022-0728(01)00343-6.

(2) Guo, J.; Lindner, E. Cyclic voltammograms at coplanar and shallow recessed microdisk

electrode arrays: guidelines for design and experiment. *Anal Chem* **2009**, *81* (1), 130–138. DOI: 10.1021/ac801592j From NLM.
(3) Shoup, D.; Szabo, A. Chronoamperometry at an ensemble of microdisk electrodes. *Journal of Electroanalytical Chemistry and Interfacial Electrochemistry* **1984**, *160* (1), 19–26. DOI: https://doi.org/10.1016/S0022-0728(84)80111-4.
(4) Mahon, P. J.; Oldham, K. B. Diffusion-Controlled Chronoamperometry at a Disk Electrode. *Analytical Chemistry* **2005**, *77* (18), 6100–6101. DOI: 10.1021/ac051034y.
(5) Aoki, K.; Tokuda, K.; Matsuda, H. Theory of stationary current-potential curves at microdisk electrodes for quasi-reversible and totally irreversible electrode reactions. *Journal of Electroanalytical Chemistry and Interfacial Electrochemistry* **1987**, *235* (1), 87–96. DOI: https://doi.org/10.1016/0022-0728(87)85199-9.
(6) Scharifker, B. R. Ensembles of Microelectrodes. In *Microelectrodes: Theory and Applications*, Montenegro, M. I., Queirós, M. A., Daschbach, J. L. Eds.; Springer Netherlands, 1991; pp 227–239.
(7) Kolev, S. D.; Simons, J. H. M.; Linden, W. E. v. d. Mathematical modelling of the chronoamperometric response of an array of-rectangular microelectrodes. *Analytica Chimica Acta* **1993**, *273*, 71–80.
(8) Komayko, A. I.; Arkharova, N. A.; Presnov, D. E.; Levin, E. E.; Nikitina, V. A. Resolving the Seeming Contradiction between the Superior Rate Capability of Prussian Blue Analogues and the Extremely Slow Ionic Diffusion. *The Journal of Physical Chemistry Letters* **2022**, *13* (14), 3165–3172. DOI: 10.1021/acs.jpclett.2c00482.
(9) Nordstrand, J.; Toledo-Carrillo, E.; Vafakhah, S.; Guo, L.; Yang, H. Y.; Kloo, L.; Dutta, J. Ladder Mechanisms of Ion Transport in Prussian Blue Analogues. *ACS Applied Materials & Interfaces* **2022**, *14* (1), 1102–1113. DOI: 10.1021/acsami.1c20910.
(10) Ly, A.; Marsman, M.; Verhagen, J.; Grasman, R. P. P. P.; Wagenmakers, E.-J. A Tutorial on Fisher information. *Journal of Mathematical Psychology* **2017**, *80*, 40–55. DOI: https://doi.org/10.1016/j.jmp.2017.05.006.
(11) Ahmad, N.; Derrible, S.; Eason, T.; Cabezas, H. Using Fisher information to track stability in multivariate systems. *Royal Society Open Science* **2016**, *3* (11). DOI: 10.1098/rsos.160582 (accessed 6/19/2026).
(12) Bouhrara, M.; Spencer, R. G. Fisher information and Cramér-Rao lower bound for experimental design in parallel imaging. *Magnetic Resonance in Medicine* **2018**, *79* (6), 3249–3255. DOI: https://doi.org/10.1002/mrm.26984.
